# Failure of the stem cell niche rather than loss of oocyte stem cells in the aging ovary

**DOI:** 10.18632/aging.100119

**Published:** 2010-01-26

**Authors:** Efi Massasa, Xavier Santamaria Costa, Hugh S. Taylor

**Affiliations:** Yale University School of Medicine, New Haven, CT 06510, USA

**Keywords:** Oocyte, stem cell, niche

The possibility of postnatal oogenesis in humans and other species has become one of today's most debated topics in the fields of reproductive and developmental biology. Wilhelm Waldeyer concluded in 1870 that oocyte production ceases with birth, and for more than a century scientists have been in consensus that the number of oocytes is gradually reduced throughout adult life [1]. A contemporary understanding of tissue regeneration, together with novel experimental methods, led to a new school of thought that has challenged the model of prenatal "total endowment", by suggesting replenishment of the postnatal oocytes pool by adult Germline Stem Cell (GSC). According to this theory, a small number of GSC remains viable to produce oocytes or oocyte-like cells within the ovary throughout the lifespan of the adult female [2,3]. These cells may originate from within or from outside the ovary. However, in a parabiotic model, or after bone marrow transplantation, the absence of mature donor derived eggs in the recipient suggested that mature eggs are not readily generated by cells from outside of the ovary [4]. Supporters of the GSC model insist that the absence of mature eggs from a donor do not contradict the idea of a progenitor cell that may give rise to a pool of additional oocytes.  Last year, Zou et. [5] established a GSC cell line by sorting for ovarian cells that were positive for the mouse vasa homologue (MVH) protein. These cells went through many passages in culture, showed high telomerase activity, and when stably transfected with GFP and transplanted into females, gave rise to offspring that carried the signal. The controversy surrounding the relation between age and the reducing number of eggs produced females only increased.

Now, in this issue of Aging, Niikura and colleagues finally clarify what had appeared to be discrepant results. They show that the premeiotic marker Stra8 and Daz1, an exclusive marker of meiosis-committed cells, are highly expressed in the ovary of aged female mice despite their being devoid of oocytes [6]. Further, by grafting an aged ovarian tissue from germ cell-specific Oct4ΔPE::GFP transgenic mice into the ovary of a young wild-type host, the authors show the formation of GFP positive immature follicles along with the co-expression of NOBOX (a primordial oocyte marker). Conversely exposure to the aged environment resulted in a reduced number of immature follicles in the young tissue. Moreover, these finding suggest that the decrease in the number of follicles is mostly due to an impaired oocyte renewal rather than an accelerated loss. This contradicts the theory of the total endowment and supports the theory of oocyte production during post-natal life.

Several important questions are left open. The first question regards the governing mechanisms that hold the immature oocyte arrested in the aging ovary, and whether these are intracellular, systematic or both. In this paper the authors demonstrate using the parabiotic model that it is not a systemic circulating factor that reduces oocyte production, rather it is a local effect suggesting the existence of a GSC niche. The failure of oocyte replenishment in the aged ovary is a result of changes in the GSC's immediate somatic environment rather than in the GSC itself.  Future studies will undoubtedly seek to identify and understand the cellular and molecular environment of the niche.

An additional question regards the potential of the GSC from aged mice to generate mature oocytes and their potential to produce progeny. Both this study and a previous study from the same group [7] showed that the oocyte-like cells formed during adult life are very immature, and are apparently limited to development to the primary stage of follicle development. It will be beneficial to perform rescue and lineage assays in order to determine whether the mechanisms that prohibit further development of premeiotic germ cells in aged ovaries are irreversible or not.

The potential clinical applications are immediately apparent.  Each year millions of women undergo fertility treatments due to age-related loss of oocytes; these treatments offer only modest improvements in fertility. By the middle of the fifth decade the therapies are largely futile. Further, millions of young women are rendered sterile by chemotherapy associated oocyte loss.  If a viable source of oocyte production remains in these women, there is potential to restore fertility. The identification of these stem cells gives hope to these women and leads us to ask if fertility restoration is possible. A further understanding and potential manipulation of the adult female germ stem cell niche may provide the answer.

## References

[1] Waldeyer W (1870). Eirstock und Ei.

[2] Johnson J, Canning J, Kaneko T, Pru JK, Tilly JL (2004). Germline stem cells and follicular renewal in the postnatal mammalian ovary. Nature.

[3] Johnson J, Bagley J, Skaznik-Wikiel M, Lee HJ, Adams GB, Niikura Y, Tschudy KS, Tilly JC, Cortes ML, Forkert R, Spitzer T, Iacomini J, Scadden DT, Tilly JL (2005). Oocyte generation in adult mammalian ovaries by putative germ cells in bone marrow and peripheral blood. Cell.

[4] Eggan K, Jurga S, Gosden R, Min IM, Wagers AJ (2006). Ovulated oocytes in adult mice derive from non-circulating germ cells. Nature.

[5] Zou K, Yuan Z, Yang Z, Luo H, Sun K, Zhou L, Xiang J, Shi L, Yu Q, Zhang Y, Hou R, Wu J (2009). Production of offspring from a germline stem cell line derived from neonatal ovaries. Nat Cell Biol.

[6] Niikura Y, Niikura T, Tilly JL (2009). Aged mouse ovaries possess rare premeiotic germ cells that can generate oocyte following transplantation into a young host environment. Aging.

[7] Lee HJ, Selesniemi K, Niikura Y, Niikura T, Klein R, Dombkowski DM, Tilly JL (2007). Bone marrow transplantation generates immature oocytes and rescues long-term fertility in a preclinical mouse model of chemotherapy-induced premature ovarian failure. J Clin Oncol.

